# Climate change could negate U.S. forest ecosystem service benefits gained through reductions in nitrogen and sulfur deposition

**DOI:** 10.1038/s41598-024-60652-z

**Published:** 2024-05-10

**Authors:** Jennifer N. Phelan, George Van Houtven, Christopher M. Clark, John Buckley, James Cajka, Ashton Hargrave, Kevin Horn, R. Quinn Thomas, Robert D. Sabo

**Affiliations:** 1 RTI International, 3040 E. Cornwallis Rd., RTP, NC USA; 2 U.S. Environmental Protection Agency, Office of Research and Development, Washington, DC USA; 3 U.S. Department of Agriculture, Forest Service, National Forest System Washington Office, Fort Collins, CO USA; 4 Department of Forest Resources and Environmental Conservation, Virginia Polytechnic Institute, Blacksburg, VA USA; 5 Department of Biological Sciences, Virginia Polytechnic Institute, Blacksburg, VA USA

**Keywords:** Ecology, Biodiversity, Climate-change ecology, Ecosystem services

## Abstract

Climate change and atmospheric deposition of nitrogen (N) and sulfur (S) impact the health and productivity of forests. Here, we explored the potential impacts of these environmental stressors on ecosystem services provided by future forests in the contiguous U.S. We found that all stand-level services benefitted (+ 2.6 to 8.1%) from reductions in N+S deposition, largely attributable to positive responses to reduced S that offset the net negative effects of lower N levels. Sawtimber responded positively (+ 0.5 to 0.6%) to some climate change, but negatively (− 2.4 to − 3.8%) to the most extreme scenarios. Aboveground carbon (C) sequestration and forest diversity were negatively impacted by all modelled changes in climate. Notably, the most extreme climate scenario eliminated gains in all three services achieved through reduced deposition. As individual tree species responded differently to climate change and atmospheric deposition, associated services unique to each species increased or decreased under future scenarios. Our results suggest that climate change should be considered when evaluating the benefits of N and S air pollution policies on the services provided by U.S. forests.

## Introduction

Climate change and atmospheric deposition of nitrogen (N) and sulfur (S) are two environmental stressors that impact the health of trees and forests and the services they provide^1–5^. Average global surface temperatures have steadily risen since the early 1900s, and although both N and S deposition in the eastern United States (U.S.) have decreased significantly since the 1990’s, deposition of the two pollutants remains 5–10 times higher than pre-industrial levels (0.4 kg N/ha/yr and 0.1 kg S/ha/yr^6,7^).

In the U.S. and elsewhere, changes in precipitation and air temperatures can influence the productivity, distribution and ranges, and phenology of individual tree species and forests^8–10^. Higher temperatures result in longer growing periods and increased decomposition and nutrient availability^11^, but also increase evapotranspiration, water stress, and pathogen and fire risk^12–17^. Similarly, altered precipitation patterns may result in repeated droughts in some areas and increased water availability in others^8^. In general, forest biomass is predicted to increase under climates with warmer temperatures^18^. However, individual tree species responses to climate vary, and while 70% of 125 tree species in the U.S. are expected to gain suitable habitat with changes in temperature and precipitation, 21% of species are forecasted to lose more than 10% of their habitat under the most extreme future climate scenarios^19^.

Nitrogen limits the productivity of many terrestrial ecosystems, and thus, increases in N deposition can increase growth and benefit forests^20–24^. However, elevated N deposition can also negatively impact tree through mechanisms including nutrient imbalances and altered competitive relationships^25–27^. Furthermore, together with S, N deposition can adversely impact trees and forests through direct foliar damage and conditions related to soil acidification^26,28, 29^. Trees differ in their sensitivity to N and S deposition. Some species increase biomass with elevated levels of N deposition, while others experience higher mortality when exposed to the same amounts of N^26^. Similarly, some tree species exhibit reduced growth while others are not impacted when exposed to soil conditions influenced by acidifying S deposition^30^.

Individually or interactively, altered climate and atmospheric deposition are therefore likely to not only result in changes in tree species abundance and distributions, but also impact the structure and function of forests. As people derive many services from trees and forests, including timber, clean air and water, and areas for enjoyment^31,32^, changes in climate and deposition can also be expected to impact valued services provided by forests.

**Figure 1:**
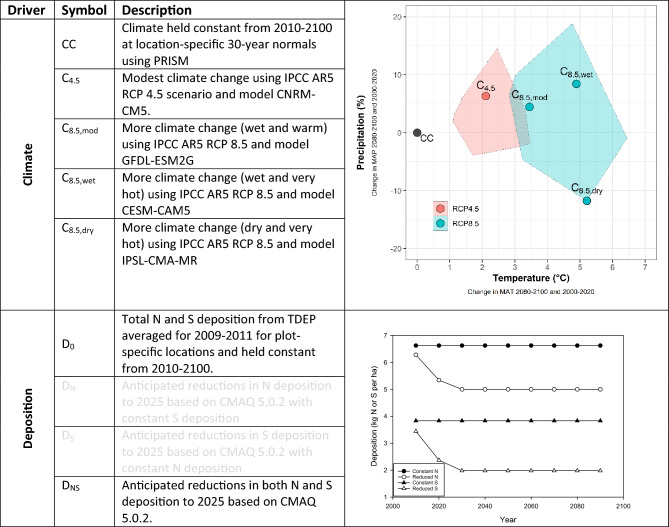
The climate and two deposition scenarios evaluated in current study. The table (left) describes the scenarios examined^33^. The climate biplot (top right) summarizes the changes between 2000-2020 and 2080–2100 in average climate conditions for the four scenarios explored in this study (points) relative to the full ensemble of IPCC AR5 models for RCP 4.5 (red polygon) and 8.5 (blue polygon). The deposition linear plot (bottom right) shows the scenarios of average deposition through time. Responses to the Constant N+S deposition (D_0_) and Reduced N+S deposition (D_NS_) scenarios were the main deposition scenarios analyzed in this study, as both N and S are anticipated to decrease with current pollution emission policies. The responses to reductions in N or S deposition individually were used to evaluate the drivers behind the responses to N+S deposition. *CC is constant climate.

Here, we evaluated the potential implications of recent climate change- and deposition-based predicted changes in forest composition^33^ on ecosystem services provided by trees and forests in the conterminous U.S. Using species-specific growth and survival models for 94 tree species that represent the vast majority of trees in most of the forested areas of the U.S., Clark and others^33^ modelled the composition of forests across the country in response to 20 different deposition—climate change scenarios out to 2100 (Fig. 1, see “Methods”). Their models included the influences of temperature, precipitation, N deposition, S deposition, tree size, and competition on individual species annual growth and decadal survival. The climate change scenarios were based on four different models and two Intergovernmental Panel on Climate Change (IPCC) Fifth Assessment Report 5 (AR5) Representative Concentration Pathway (RCP) emission scenarios to represent a range of potential climate futures. The deposition scenarios were based on Clean Air Act (CAA) policies and emission reductions^34^ and included current deposition held constant and anticipated reductions in N, S, and N+S deposition. In a parallel 2019 study, Van Houtven and others used a comparable, but more limited set of models for 24 tree species in 19 states^1^ to predict forest responses to climate scenarios and reductions in N deposition in the northeastern U.S. However, they extended their analyses to also determine how associated stand-level ecosystem services were also impacted by potential climate futures and reductions in N. More recently, Cavender-Bares and others^32^ quantified regulating and provisioning stand-level ecosystem services by tree lineages of 400 species across the contiguous U.S. in 2010–2012, but noted the uncertainty and the importance of modeling services into the future under threats of climate change, fire, pests, and pathogens. The impacts of a future changing climate and policy-based reductions in air pollution on the services provided by forests in the U.S. have therefore not been fully explored and represent an important information gap.

Building off the nationwide forest composition projects from Clark and others^33^, the recommendations of Cavender-Bares and others^32^, and the methodology piloted in the northeastern U.S. by Van Houtven and others^2^, we determined how four different climate change scenarios and policy-based reductions in N and S deposition could influence the services provided by future stand-level forests and 94 individual tree species in the contiguous U.S. More specifically, we went beyond previous work and evaluated the impacts of climate and deposition on aboveground carbon (C) sequestration and associated economic value, sawtimber volume and value, and forest diversity at the stand level, and on the provisioning, cultural, and unique services offered by each tree species out to 2100. Forest tree diversity at the stand-level was determined using the Shannon Weaver (H) Index of Diversity^35^ converted to Effective Number of Species^36^ (see “Methods”). “Unique service” represents an ecosystem service that is not easily substituted and is only offered by one or a few tree species. All analyses and comparisons between scenarios were conducted at the national, contiguous U.S. level to gain an understanding of the net impacts of potential climate and deposition futures on the various services collectively offered by the nation’s forests. Analyses of the regional variation in responses of each service across the U.S. are available in a recent publication^37^ or are currently underway.

## Results and discussion

### Forest stand-level services

Changes in climate and reductions in N+S deposition were projected to have significant impacts on each of the forest stand-level ecosystem services. We found decreased N+S deposition (D_NS_) consistently increased the amounts and economic values of all three services, while climate change, especially the most extreme climate change scenarios (C_8.5,wet_ and C_8.5,dry_), almost always reduced the services provided by forests in the contiguous U.S. in 2100 (Table 1).

**Table 1:** Summary of forest stand-level ecosystem services in 2100 in response to the deposition—climate change scenarios.

Scenario	Sequested aboveground carbon (C)				Sawtimber				Effective number of species (ENS)	
	Total—thousand Mt (rank^a^)	Percent reduction^b^ in Total	Present value^c^—trillions $ (rank)	Percent reduction in Value	Total volume million MBF (rank)	Percent reduction in volume	Total value—trillions $ (rank)	Percent reduction in value	Average number (rank)	Percent reduction in average number
constant N+S deposition/constant climate (D_0_/CC)	14.70 (4)	− 7.5%	$1.47 (4)	− 6.6%	26.45 (7)	− 4.0%	$3.61 (6)	− 2.5%	7.47 (2)	− 2.4%
constant N+S deposition/modest climate change (D_0_/C_4.5_)	14.20 (5)	− 10.6%	$1.44 (5)	− 8.4%	26.6 (6)	− 3.5%	$3.63 (4)	− 1.9%	7.27 (5)	− 5.0%
constant N+S deposition/wet and warm climate (D_0_/C_8.5,mod_)	13.78 (6)	− 13.2%	$1.42 (7)	− 9.9%	26.64 (5)	− 3.3%	$3.62 (5)	− 2.2%	7.17 (8)	− 6.3%
constant N+S deposition/wet and very hot climate (D_0_/C_8.5,wet_)	12.48 (9)	− 21.4%	$1.34 (9)	− 15.3%	25.86 (9)	− 6.1%	$3.50 (9)	− 5.5%	7.00 (9)	− 8.5%
constant N+S deposition/dry and very hot climate (D_0_/C_8.5,dry_)	*12.11 (10)*	*− 23.7%*	*$1.31 (10)*	*− 17.1%*	*25.51 (10)*	*− 7.4%*	*$3.48 (10*)	*− 6.0%*	*6.99 (10)*	*− 8.6%*
reduced N+S deposition/constant climate (D_NS_/CC)	**15.88 (1)**		**$1.58 (1)**		27.42 (3)	− 0.5%	$3.69 (3)	− 0.5%	**7.65 (1)**	
reduced N+S deposition/modest climate change (D_NS_/C_4.5_)	15.33 (2)	− 3.5%	$1.55 (2)	− 2.1%	27.53 (2)	− 0.1%	**$3.70 (1)**		7.46 (3)	− 2.5%
reduced N+S deposition/wet and warm climate (D_NS_/C_8.5,mod_)	14.88 (3)	− 6.3%	$1.52 (3)	− 3.7%	**27.56 (1)**		$3.69 (2)	− 0.4%	7.36 (4)	− 3.8%
reduced N+S deposition/wet and very hot climate (D_NS_/C_8.5,_wet)	13.51 (7)	− 14.9%	$1.43 (6)	− 9.3%	26.73 (4)	− 3.0%	$3.56 (7)	− 3.8%	7.19 (6)	− 6.0%
reduced N+S deposition/dry and very hot climate (D_NS_/C_8.5,dry_)	13.11 (8)	− 17.5%	$1.40 (8)	− 11.4%	26.34 (8)	− 4.4%	$3.54 (8)	− 4.4%	7.18 (7)	− 6.1%

For each service, italic and bold values indicate the scenarios with the smallest and largest amount of service provided in 2100, respectively.

^a^“Rank” indicates the order of results from highest to lowest for each service.

^b^^“^Percent reduction” indicates the percent reduction in the service relative to the #1 ranked service in 2100.

^c^“Present value” of sequestered C represents the reduced SCC from all modeled current and future sequestration (up to 2100), discounted to 2015 at a 3% discount rate.

For sequestered C and forest diversity, N+S reductions with a constant, non-changing climate (D_NS_/CC) produced the largest amounts and values of services, while constant N+S combined with a dry and very hot climate (D_0_/C_8.5,dry_) resulted in the lowest of both (Table 1). The largest difference between the two contrasting scenarios occurred with total C sequestered, representing a total of 3,769 Mt of C and $271 billion of potentially lost value of C in 2100. Forest diversity experienced the lowest impacts with effectively only 1 species difference between scenarios. However, Shannon Weaver (H) is a conservative diversity index, and more sensitive diversity indices may have resulted in larger estimates of change^38^.

In contrast, although the lowest sawtimber volumes and values also occurred with constant N+S deposition combined with the most extreme changes in climate (D_0_/C_8.5,dry_), sawtimber responded most favorably to reduced N+S and some climate change (Table 1). We found the largest sawtimber responses occurring with N+S reductions combined with a moderately altered, wet and warm climate (D_NS_/C_8.5,mod_) for sawtimber volume and a modest change in climate (D_NS_/C_4.5_) for sawtimber values, representing predicted differences of 2.045 million metric board feet (MBF) and $224 billion between the contrasting scenarios in 2100. The differences in sawtimber volume and value responses were due, at least in part, to the relatively more valuable sawtimber species responding overall less favorably to the wet and warm climate (C_8.5,mod_). These positive responses of sawtimber to some climate change were somewhat surprising as sawtimber volume, like forest C, is a function of total forest biomass, which consistently responded negatively to all four climate change futures (Table 1)^33^. Potential explanations for this deviation in sawtimber volume response to climate change include the assemblage of sawtimber species analyzed and the methodology employed. All tree species sequester C, but not all tree species are considered sawtimber. Sawtimber volume, in our study, disproportionately consisted of species such as Douglas fir (*Pseudotsuga menziesii,* 21% of sawtimber, on average, among scenarios in 2100) that responded favorably to all climate change scenarios, and did not include relatively abundant species such as sweetgum (*Liquidambar styraciflua*), slash pine (*Pinus elliottii*), balsam fir (*Abies balsamea*), and black walnut (*Juglans nigra*), which responded negatively to the four climate change scenarios that we assessed. In addition, sawtimber volumes and values were based on sales transactions from USFS lands by USFS National Forest region (Fig. S1), and the USFS lands may not include all forest types and sawtimber species present and harvested on private and forest industry lands within each region. For example, red maple (*Acer rubrum*) responded positively to climate change in Region 9, but negatively to all four climate scenarios in Region 8. However, sawtimber volumes and sales were only reported for USFS lands in region 9 (Fig. S1), thereby biasing and inaccurately representing the influences of climate on sawtimber estimates for red maple that represented, on average, 6% of sawtimber in 2100. As an estimated 56% of all forested land in the U.S. is privately owned, and the National Forest lands managed by the USFS only represent 33% of the nation’s forests^39^, a revised analysis that also includes sales from private and industry lands is therefore warranted and may result in different projected trends in sawtimber volumes and values in response to future climates. Such analyses may be particularly important in areas of the eastern U.S. where a larger proportion of forests are privately owned (https://experience.arcgis.com/experience/82dcef460b1a470db0f8f4dd7cf6f9b7/page/page_3/).

**Figure 2:**
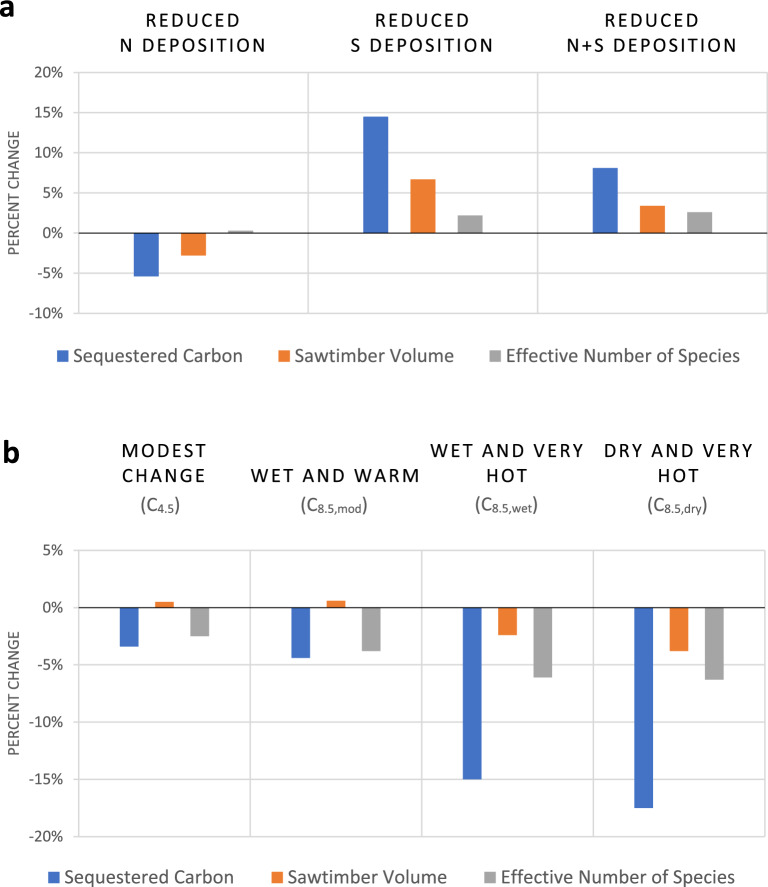
Average influences of the forms of deposition (**a**) and four climate change scenarios (**b**) on the forest stand-level ecosystem services (relative to constant deposition (D_0_) or constant climate (CC)). See Table S1 for results for individual scenarios.

We next explored the impact of individual drivers behind the ecosystem service responses to reduced N+S deposition and climate change (Fig. 2a,b, Table S1). For the three stand-level services, we found the positive responses to reductions in N+S atmospheric deposition were attributable largely to S. Reduced S deposition accounted for 2.2% to 14.5% of the increases in sequestered C, sawtimber volume, and forest diversity. As S is a main component of acid rain^40^, reduced S deposition would ultimately result in decreased losses of base cations and subsequent reductions in soil acidification, thereby benefitting acid-sensitive tree species and the services they provide. Multiple studies have reported improved tree health and growth when acidic soil conditions are alleviated^41–43^. However, the natural recovery of ecosystems from acidification is often delayed^44,45^, so the timing and magnitude of ecosystem service responses to reductions in S deposition in our study may be overestimated in some of the forests.

Contrasting the responses to S, we found reductions in N deposition resulted in indiscernible changes or decreases (0.3% to − 5.4%) in the three stand-level ecosystem services evaluated, partly offsetting but not eliminating the gains achieved with reductions in S. Nitrogen commonly limits the growth in terrestrial ecosystems^20–24^, and fertilizer trials and silvicultural practices have documented improved growth of trees with additions of N^46–48^. Therefore, although some species modelled by Clark and others^33^ increase growth and survival at lower levels of N, when evaluated at the forest stand level summarized for the conterminous U.S., decreased N deposition could be expected to result in net lower productivity and reductions in the services provided by forests.

We were unable to isolate the influences of temperature versus precipitation in our study because changes in both drivers co-occurred in the IPCC AR5 climate scenarios. Nevertheless, the patterns of response to the progressively more extreme changes in climate, especially temperatures (+ 2 to + 5 °C), were generally consistent (Table 1). All four climate change scenarios reduced C sequestered and forest diversity, with negative impacts increasing with severity of the climate change scenario. The percent decrease in these two services ranged from − 2.5 to − 3.4% for modest changes in climate (C_4.5_) to − 6.3% to − 17.5% for the most extreme changes to dry and very hot (C_8.5,dry_) conditions (Fig. 2a,b, Table S1). For sawtimber, both the modest change (C_4.5_) and moderate, wet and warm (C_8.5,mod_) climate conditions resulted in small but positive increases (0.5% and 0.6%, respectively), but the two most extreme climate scenarios with the hottest temperatures (C_8.5,wet_, C_8.5,dry_), decreased sawtimber volume by − 2.4% and − 3.8%, respectively, indicating that sawtimber species are negatively impacted by more dramatic changes in temperature. Similar results were reported by Baker and others (2022), who evaluated the impacts of six RCP 8.5 climate scenarios out to 2095; cumulative harvested timber (sawtimber and pulpwood) was reduced under all but one of the scenarios, with the hottest temperature scenarios resulting in 3 to 6% declines in cumulative harvest relative to the constant climate future. However, for all three stand-level services in our study, wetter average conditions partially mitigated the effects of higher temperatures, as indicated by the 0.2 to 2.5% differences in the responses to the dry (C_8.5,dry_) vs. wet (C_8.5,wet_) and very hot scenarios.

Some climate-sensitive species were projected to be lost entirely at the state-level (Table S2), though not lost nationally, supporting hypothesized^50,51^ and observed^52–54^ climate-induced changes in suitable habitat and shifts in species ranges. By 2100, in response to all climate scenarios, sassafras (*Sassafras albidum*) was eliminated from four states (MS, NH, OK, TX), Jack pine (*Pinus banksiana*) from two (NE, NY), and black oak (*Quercus velutina*) from one (TX). Even though each species only accounted for a small proportion of total forest biomass in each state (all < 2% of 2010 total forest biomass), these projected species losses likely accounted for at least some of the sensitivity of the forest diversity service to climate change.

Lastly, with the opposing influences of reduced N+S deposition and changes in climate on forest stand-level services, we wanted to further evaluate if, and under what conditions, changes in climate counter or eliminate the benefits in services gained through reductions in deposition. Through a comparison of the individual scenario responses, we found that the benefits of lower N and S deposition were completely offset by some climate futures (Table 1, using the D_NS_/CC as the reference scenario). All climate change scenarios eliminated the benefits of reduced N+S deposition on forest diversity, and the two most extreme temperature scenarios (C_8.5,wet_, C_8.5,dry_), countered the gains of reduced N+S deposition on sequestered C. Although the scenarios with the smallest changes in climate (i.e., C_4.5_ and/or C_8.5, mod_) further supplemented the positive sawtimber volume and value responses to reduced N+S deposition, indicating that some services provided by forests may benefit from a combination of reduced N and S emissions and changes in precipitation and warmer temperatures, more extreme dry and very hot climate futures (i.e., C_8.5,dry_) completely negated all increases in sawtimber volume and value achieved with reduced N+S. These results, therefore, indicate that some, especially the most extreme, climate futures have the potential to fully counter and essentially eliminate the gains in some forest stand-level services achieved with policy-based reductions in N+S deposition.

### Individual species services

To gain a better understanding of the impacts of future climate and deposition on services often not captured at the stand level, we also examined ecosystem services provided by individual tree species^55^. These included services such as high-quality wood for musical instruments (e.g., from red spruce—*Picea rubens*), sap for syrup (e.g., from paper birch—*Betula payrifera*), and resins used in a variety of personal care and other products (e.g., from slash pine—*Pinus elliottii*). We evaluated both the total number of provisioning and cultural services (Table S3), and the services unique to individual species.

Using the U.S. Forest Service (USFS) FEIS database^55^, we found a total of 449 overlapping provisioning and cultural services are provided by the 94 tree species included in our assessment (Table S4). Because each species differs in their sensitivities to reductions in N+S deposition and climate change^33^, we found some services were positively impacted by changes in both drivers, others negatively impacted by both, and the rest were a mixture of positive and negative responses (Table 2). Consistent with the forest stand-level services, most tree species and their respective services (44 species and 189 services) were impacted positively by reductions in deposition and negatively by all changes in climate, with the changes in climate negating the benefits of reduced deposition for 27 of 44 tree species (and 126 of the 189 services) (Table S4). A small proportion of the species and associated services (10 species and 53 services) were negatively impacted by only certain climate scenarios, mainly the most extreme scenarios with the hottest temperatures (C_8.5,wet_, C_8.5,dry_). Although this summary suggests that ecosystem services offered by individual tree species are likely to be reduced with the potential N+S deposition and climate futures evaluated in our study, many of these services are provided by multiple species. Therefore, reductions in services provided by species adversely impacted by the future conditions may be replaced by surrogate species that benefit from the conditions. For example, high-quality wood supplied by black cherry (*Prunus serotina*) could potentially be substituted by sugar maple (*Acer saccharum*), as both woods are used in furniture manufacturing, and while black cherry was projected to respond negatively, sugar maple responded positively to reduced N+S deposition and the climate futures^33^.

**Table 2:** Summary of the responses of the 94 individual tree species and associated provisioning and cultural services to reduced N+S deposition and climate change scenarios.

Response to reduced N+S deposition	Response to climate change	Count of species	Proportion of total forest biomass (2010)—%	Count of provisioning and cultural services
+	+	14	35	74
	−	44	27.7	189
	mixed (+ or −)	9	13.2	49
−	+	7	7.4	39
	−	19	15.8	94
	mixed (+ or −)	1	0.8	4

“+” indicates positive response of individual tree species.

“−” indicates negative response of individual tree species.

“mixed (+ or −)” indicates a positive response of individual tree species to some scenarios and negative response to the others.

At the same time, not all ecosystem services can be easily substituted by alternate tree species. Tables S5 and S6 provide summaries of select species and associated unique services that are only offered by a single or a limited number of species and are projected to be positively (Table S5) and negatively (Table S6) impacted by the reductions in N+S deposition, respectively, across climate change scenarios. For example, red spruce (*Picea rubens*), a species that has been negatively impacted by acid deposition^56^, was modelled to be positively impacted by both reduced N+S deposition and all four climate change futures^33^. Therefore, services from red spruce, including timber production and musical instruments^57^, are projected to benefit under such future conditions. In contrast, species such as sassafras (*Sassafras albidum*) and pitch pine (*Pinus rigida*) were forecasted to be positively impacted by N+S deposition reductions, but these benefits were offset by the negative effects from climate change. Thus, services including oils for flavoring and perfumes^58^ and habitat for the endangered Karner blue butterfly^59^ would be reduced under all combined deposition and climate futures that we evaluated. Arguably, of greatest concern, are the services provided by the tree species that responded negatively to both reductions in deposition and climate change. There were 19 species in this category (Table 2; Table S4), including longleaf pine (*Pinus palustris*) that provides habitat for endangered red cockaded woodpecker^60^, black walnut (*Juglans nigra*) that supplies high quality, unique-grained wood for furniture^61^, and swamp tupelo (*Nyssa biflora*) that serves as a source of nectar to produce honey^62^. Such services could be significantly reduced in the future. Given the variation of tree species responses to reduced N+S deposition and changes in climate, combined with the large number and variety of provisioning, cultural, and unique ecosystem services offered by individual tree species, it is clear that more sophisticated analyses of ecosystem services that move beyond aggregate functions like C and clean water are necessary. The degree to which services can be replaced by surrogate species is likely to vary by type of service, region, forest attributes, mill capacity, supply and demand, and other conditions. Similarly, there are unique and highly valued services offered by individual tree species expected to be impacted by future N+S deposition and/or climate conditions that could be lost or significantly reduced. Both merit further, more detailed study.

## Limitations

There are several limitations in our ecosystem service evaluation that contribute to uncertainty in the findings and suggest areas of future research. First, the forest composition projections from Clark and others^33^ were for the current, single cohort of non-intensively managed, mixed-age and mixed-species trees detailed in the FIA database. Thus, our projections do not include the influences of ingrowth or recruitment of new individuals or silvicultural interventions such as planting, thinning, or harvesting which could affect forest composition and associated ecosystem services in 2100. Second, only the more abundant tree species are presented in the projections, and although these represent a large fraction of the contemporary forest (> 90% of the basal area in most of the contiguous U.S.), rarer species were not captured, and dominance by the existing cohort of species may change in the future. Third, predicted deposition, temperature, and precipitation (corresponding to the climate change scenarios) in some locations were outside the range of data used to produce some of the tree species growth and survival models. A total of 1%, 6%, 19%, and 20% of trees experienced high temperatures beyond their modelled temperature ranges for the C_4.5_, C_8.5,mod_, C_8.5,wet_, and C_8.5,dry_ scenarios, respectively, and it is uncertain how tree growth and survival, and the resulting forest composition and associated ecosystem services, respond outside the modelled conditions. Fourth, although temperature, precipitation, atmospheric deposition, competition, and size are strong determinants of tree growth and survival, there are dozens of other influential factors that could affect our results. Of particular importance to this study are unaccounted for climate-related factors including CO_2_ concentrations and episodic events such as drought, frost, pest outbreaks, and wildfires. For example, Thomas and others^63^ predicted enhanced growth of loblolly pine (*Pinus taeda*) in response to elevated CO_2_ to be greater than reductions in growth associated with changes in temperature or precipitation. Loblolly pine is a main sawtimber species and accounts for 7% of sawtimber volume in our study. Similarly, Chambers and others^64^ reported reduced regeneration of ponderosa pine (*Pinus ponderosa*) following severe wildfires, and wide-spread mortality of piñon (*Pinus eludis*) and ponderosa pine have been attributed to drought-related reductions in host tree resistance to outbreaks in two species of pine beetles^65^. Pests and pathogens are estimated to threaten 16% of tree species, potentially impacting up to 40% of total tree biomass in the U.S., and increased fire frequency is expected to impact 40% of U.S. tree species^32^. Expanding the species-specific growth and survival models to include some of these additional climate-related factors and other influential factors such as soil conditions and mycorrhizal associations is an ongoing and important area of research. Finally, our analyses focused on the net impacts of future climate and atmospheric deposition on ecosystem services offered by U.S. forests at the national level. A summary at this level provides an indication of the role and importance of U.S. forests in C sequestration, timber production, and biodiversity into the future. However, services summed for the whole U.S. do not recognize the variability of individual species and stand-level responses to climate and deposition and that some areas see large gains in services while others see loses. It is beyond the scope of this current study to evaluate the regional differences in the ecosystem services provided by forests. Instead, we point readers to Reese and others^37^ that documents the variability and regional responses of sequestered C to the climate and deposition futures. Similar detailed regional analyses of the responses of sawtimber, pulpwood, and forest diversity to the future conditions are currently underway.

## Conclusions

Here, we have explored the potential impacts of changes in precipitation and temperature and reductions in N+S deposition on the ecosystem services provide by future forests in the contiguous U.S. We found that all forest stand-level services benefitted from reductions in N+S deposition. These trends were largely attributable to positive responses to reduced S deposition that offset the potentially negative effects of lower levels of N, thereby indicating the net benefits of air quality policy on the services provided by the nation’s forests. We also found for two out of the three services (C sequestration and biodiversity), all the modelled changes in climate negatively impacted the amount and value of services provided, with the most extreme scenarios with the hottest temperatures completely eliminating the gains achieved through reduced deposition. Although sawtimber responded favorably to some climate change, it too was negatively impacted by the most extreme climate change scenarios. Ecosystem services unique to individual tree species were found to be a mixture of positive and negative responses. Given these results and the predictions that some climate futures have the potential to fully counter gains associated with reductions in N+S deposition, policy makers and natural resource managers should consider climate change when evaluating the benefits of N and S air pollution policies on the stand-level and individual tree species services provided by U.S. forests.

## Methods

### Forest composition

The forest composition and tree aboveground biomass estimates in 2100 were taken directly from Clark and others^33^. The following sections briefly describe the tree database, model, and climate and deposition scenarios used to produce these estimates.

#### Tree database

Briefly, Clark and others^33^ assembled a tree database from the U.S. Forest Service Forest Inventory and Analysis (FIA) program. This dataset consisted of tree and plot (fuzzed coordinates) data for 124,431 plots, 352 species, and 2,851,772 individual trees across the conterminous U.S. Expanded to the county-level, the database represented a total of 19,293,749,019 trees and 34,347,704,676 metric tonnes of above-ground biomass. Although the tree data were for conditions measured in 2000–2016, all trees were assumed to be their reported FIA biomass in the model start year of 2010 for consistency with the future deposition and climate scenarios.

#### Model

The species-specific growth and survival equations described by Clark et al.^33^ were applied to the trees in the Tree Database. Only species with ≥ 2000 records (i.e., 94 of the 352 tree species in the database) were modelled. However, these 94 species were found on 120,159 of the 124,731 FIA plots (i.e., 96.3%) in the Tree Database, and on average, represented 93.2% of plot basal area. The Tree Database served as the source of the starting tree and sub-plot basal area model input data, and the deposition and climate scenarios (described below) were the sources of the plot-level precipitation, temperature, and N and S deposition estimates. All model input data were represented at the FIA plot- or individual tree-level. Non-modelled tree biomass was included and held constant at the 2010 levels throughout the simulations. Only a single cohort of trees were modeled. Ingrowth of new trees was not included, but efforts are underway to model the impacts of deposition and climate change on recruitment of seedlings.

The forest composition model estimated changes in above-ground biomass (i.e., growth) and survival in 10-year time steps, from 2010 to 2100, in response to each of the 20 deposition—climate scenarios. For each time step, individual tree growth and survival (for the 94 species) were modeled at the sub-plot level, with individual tree biomass and proportion of surviving individuals at the end of the previous time step serving as the starting conditions for the next time step. Individual tree growth was modelled as an annual rate multiplied by 10. Individual tree survival was modelled as a 10-year probability. Only modelled trees grew and died. The remaining 258 non-modelled tree species were included, but held constant at 2010 aboveground biomass throughout the model simulations. To prevent forecasting growth and survival beyond modelled conditions, the growth and survival estimates were restricted to the observed ranges of the data used to produce the relationships. Once plot conditions were outside the training data ranges for a species, annual growth and survival were modeled to continue at the rate associated with the species-specific upper or lower limit for that parameter. Likewise, to prevent individual trees from growing beyond observed sizes, all trees were modeled to stop growing (i.e., individual tree growth of a species was set to 0 kg/yr) once they reached the largest recorded biomass for their species (i.e., biological maximum) within the USFS FIA database. It should be noted that these restrictions may have resulted in conservative growth and survival predictions, as trees are likely to respond more adversely as conditions such as higher temperatures continue to increase beyond the range currently experienced by the trees.

At the end of each times step, the biomass and probability of survival of each tree in each FIA plot was expanded to the county-level using FIA plot-specific expansion factors to represent full forest coverage (stem counts and total biomass) of each species in each county. These county-level values were then summed at the national level to represent individual species and forest biomass responses to the 20 deposition—climate scenarios. Predicted survival rates resulted in some trees being reduced to less than 1 individual at the county level. Similarly, very low trees per hectare counts (approximate 0.00025 trees per ha) for an individual tree resulted in some survival equation predictions returning an “error”. In these situations, the tree was recorded as dead, thereby representing 0 trees per hectare at the county level in the next model time step.

See Clark et al.^33^ for additional information regarding the application of the growth and survival models at the FIA plot level to produce the national tree species and forest biomass estimates.

#### Deposition and climate scenarios

Total Deposition (TDEP) served as the source of the N and S deposition estimates for the tree growth and survival models used by Clark and others^33^. Future deposition scenarios were from CMAQ v5.0.2^66^ total N and S deposition estimates applied as scaling factors (determined for each plot) to TDEP to estimate reductions in deposition associated with the policy-based reductions in emissions (Fig. 1).

*Current Constant* - this scenario represented 2010 deposition levels maintained over time and consisted of plot-specific 3-year average (2009–2011) TDEP N and S deposition repeated each year from 2010 to 2100.

*CAA 2028–N* - this scenario represented policy-based reductions in N deposition while maintaining S deposition at 2010 levels. Reductions in total N deposition were based on the differences in the two years (2011 and 2028) of CMAQ total N deposition converted into plot-specific % changes in deposition. These % changes in deposition were then applied to the plot-specific TDEP “constant” N deposition, as linear declines in annual TDEP N deposition from 2011 to 2028. Total N deposition in 2028 served as the estimate of annual N deposition from 2029 to 2100. Total S deposition remained at the 2010 “constant” level for the full simulation (2010–2100).

*CAA 2028–S* - this scenario represented policy-based reductions in S deposition while maintaining N deposition at 2010 levels. Deposition for this scenario was identical to the CAA 2028–N scenario except reductions in total S deposition were estimated, while N deposition remained at the 2010 “constant” level for the full simulation.

*CAA 2028–N*+*S Decrease* - this scenario represented policy-based reductions in both N and S deposition. Deposition for this scenario consisted of reductions in N and S deposition estimated for the CAA 2028–N and CAA 2028–S deposition scenarios (with 2028 N and S deposition being repeated from 2029 to 2100).

The climate scenarios used by Clark and others^33^ included estimates of total annual precipitation and average annual temperature. PRISM (http://www.prism.oregonstate.edu/) served as the source of temperature and precipitation estimates for each plot. The future climate scenarios were based on four different models and two IPCC Representative Concentration Pathway (RCP) emission scenarios (Fig. 1) that have been statistically downscaled as Localized Constructed Analogs (LOCA) datasets (https://journals.ametsoc.org/doi/pdf/10.1175/JHM-D-14-0082.1) to represent the ranges of potential temperatures and precipitation futures (Fig. 1). Changes in climate associated with each of these future scenarios were applied as scaling factors (determined for each plot) to the PRISM data to estimate changes in annual temperature and precipitation predicted by the future scenarios (Fig. 1).

*Constant* - this scenario represented recent climate maintained over time and consisted of plot-specific 30-year average (1981–2010) 4-km PRISM temperature and precipitation estimates repeated from 2010 to 2100.

*RCP Climate Scenarios* - these scenarios represented four potential climate futures from two of the IPCC RCP emission scenarios (RCP 4.5 and 8.5). For each of the four climate scenarios, the differences between 10-year averages (2006–2015 and 2090–2099) of the modelled precipitation and temperature estimates were used to calculate the annual percent (precipitation) or amount (temperature) of change at each plot. These changes were then be applied to the plot-specific PRISM “constant” climate conditions, as linear decreases or increases in average temperature and precipitation from 2010 to 2100.

Total annual N and S deposition, annual precipitation, and average annual temperature for the deposition and climate scenarios were converted into 10-year averages (2010–2019….2080–2089). These 10-year deposition, precipitation, and temperature averages served as the 20 deposition—climate scenario input data for the Clark and others^33^ growth and survival models and forest composition estimates.

See Clark and others^33^ for additional information regarding the selection of the deposition and climate scenarios included in the model simulations. Clark et al.^33^ also describe the varied responses to the regional patterns of precipitation, temperature, and deposition across the conterminous U.S. that locally influence the growth and survival of the modelled trees.

### Ecosystem services

#### Forest carbon sequestration

The tree biomass estimates from the forest composition analysis model were used to calculate total aboveground forest C (metric tonnes) in the study area for each 10-year time step from 2010 to 2100. Calculations were conducted at the county level and summed to the 48-state study area. A 50% C content factor was used to convert total biomass to C^26^.

Changes in stored C (i.e., C sequestration—for each 10-year time step from 2010 to 2100) were calculated and then used to estimate the total social value of each increment. For valuation, the change in C was multiplied by $-per-tonne social cost of carbon (SCC) values reported in the Interagency Task Force report^67^ on SCC for each 10-year period. The selected SCC estimates from the report were derived using a central assumption of a 3% annual discount rate. All these values were inflation-adjusted (using the GDP-deflator price index) to 2015 dollars (https://apps.bea.gov/iTable/index_nipa.cfm). The year 2015 was selected as the reference period because it is the mid-point of the first 10-year model period.

Lastly, for each of the 20 scenarios, the estimated values for each 10-year time step were combined to estimate the discounted present value (in 2015) of C sequestration for all modeled tree species over the entire study period. In other words, the economic (SCC-based) value estimates for total modeled C sequestration were summed together in each time step, with the values occurring farther in the future being down-weighted in this summation using the principles of economic discounting (i.e., using 2015 as the reference period and the 3% annual discount rate described above).

#### Sawtimber

The following “total merchantable value” (TMV) index was developed to represent sawtimber values for each scenario *j*

1 $${{\text{TMV}}}_{j}={\sum }_{i=1}^{N}{P}_{i}*{V}_{ij}$$

where *N* is the number of sawtimber species, *P*_*i*_ is the average stumpage price (in $/MBF) for tree species *i*, and *V*_*ij*_ is the modeled volume of the standing stock (in MBF) of the sawtimber species *i* under scenario *j*.

In essence, TMV is a price/value-weighted sum of the modeled tree biomass (converted to volume using the method described in Van Houtven et al.^2^) for all *N* species combined. As can be seen in Eq. (1), the species’ price (*P*_*i*_), in effect, magnifies the effect of a change in its volume (*V*_*i*_) on TMV. Therefore, a change in the volume of higher priced species has a larger proportional effect on TMV than the same change in a lower priced species. Consequently, If TMV increases by more [less] than total tree volume of all species combined, then the composition of this total tree volume must have shifted towards trees with generally higher [lower] values (price).

Stumpage price estimates were based on data from the U.S. Forest Service (USFS) cut-and-sold reports from 2014 to 2018 (https://www.fs.fed.us/forestmanagement/products/cut-sold/index.shtml). This database tracks all sales of forest products harvested from National Forest System lands, with separate records for sawtimber and pulpwood sales. For each transaction, it reports the geographic location, date, tree species, and forest product sold, as well as the total volume (in MBF) and total value (in $s) of the sale. A stumpage price for each transaction was estimated by the ratio of total value to total volume of the sale. To account for spatial variation in prices across the U.S., average (volume-weighted) transaction prices for each species were separately calculated for each of the eight USFS National Forest regions. Outlier values and average price estimates based on fewer than 10 transactions were dropped from the data. For species that could not be exactly matched to the species names reported in the cut-and-sold transactions data, average prices for the most closely corresponding species group, e.g., “oak” vs. “scarlet oak”, were used as proxies, when available.

The designation of individual tree species as sawtimber species was done separately for each forest region and depended on whether at least 10 transactions for the species were labeled as sawtimber sales in the cut-and-sold reports for the region. Therefore, a tree could be considered sawtimber in one region and not another. The percent of total modeled tree volume that was designated as sawtimber varied across regions, but averaged 84% for the entire contiguous U.S. across the 20 scenarios.

Combining price and modeled volume estimates, TMVs were estimated for each scenario and region in 2100. National estimates of TMV were estimated by summing across all regions.

#### Tree species diversity

Tree species diversity was estimated using the Shannon–Weaver Diversity (S) Index:

$${{\text{S}}}_{ij}={\sum }_{j=1}^{{n}_{i}}{p}_{ij}*{\text{ln}}({p}_{ij})$$

where *p*_*ij*_, proportion of total tree biomass in location *i* (i.e., county or region) that is contributed by tree species *j*; *n*_*i*_, number of tree species (from the set of 94 species included in the study) in location *i.*

Diversity index values were calculated at the county level using the 2100 above-ground biomass results for the 94 modelled tree species in response to the 20 deposition–climate scenarios. The S values were then converted to Effective Number of Species (ENS) estimates:

$${\text{ENS}}={e}^{{\text{S}}}$$

where S, Shannon–Weaver Diversity Index.

### Supplementary Information

Supplementary Information.

## Data Availability

The datasets generated during and/or analysed during the current study are available in the Supplementary Materials or from the corresponding author on reasonable request.
