# Supplementary material for: Climate change could negate U.S. forest ecosystem service benefits gained through reductions in nitrogen and sulfur deposition
Source: Sci Rep. 2024 May 10;14:10767. doi: 10.1038/s41598-024-60652-z (PMC11087459; doi:10.1038/s41598-024-60652-z)
Supplement: Supplementary file 1 — Supplementary Information. [file 41598_2024_60652_MOESM1_ESM.docx]

**Supplemental Materials**

**Table S1.** Influences of the forms of deposition and four climate change scenarios on the forest stand-level ecosystem services. The service amounts are calculated as the difference between the scenarios averaged for each deposition form or climate change scenario and constant deposition or climate (e.g., deposition form (D_N_, D_S_, D_NS_ – constant deposition (D_0_)). The percentages are the percent differences calculated using the services averaged by deposition form and climate scenario and (deposition or scenario – constant)/constant.

| **Driver** | **Model type** | **Sequestered Carbon** | | **Sawtimber Volume** | | **Effective Number of Species** | |
| --- | --- | --- | --- | --- | --- | --- | --- |
|  |  | **(Mt)** | **(%)** | **(MBF)** | **(%)** | **#** | **(%)** |
| Deposition | N reduction (D_N_) | -720 | -5.4% | -725,769,479 | -2.8% | 0.02 | 0.3% |
|  | S reduction (D_S_) | 1,950 | 14.5% | 1,769,396,782 | 6.7% | 0.16 | 2.2% |
|  | N+S reduction (D_NS_) | 1,089 | 8.1% | 900,842,495 | 3.4% | 0.19 | 2.6% |
| Climate Change | Modest climate change (C_4.5_) | -526 | -3.4% | 127,515,758 | 0.5% | -0.19 | -2.5% |
|  | Wet and warm climate (C_8.5,mod_) | -678 | -4.4% | 163,223,701 | 0.6% | -0.29 | -3.8% |
|  | Wet and very hot climate (C_8.5,wet_) | -2,297 | -15.0% | 642,343,366 | -2.4% | -0.46 | -6.1% |
|  | Dry and very hot climate (C_8.5,dry_) | -2,683 | -17.5% | 1,013,591,590 | -3.8% | -0.48 | -6.3% |

**Table S2.** Species eliminated in at least 1 state in at least 1 scenario by 2100; some species eliminated with all scenarios (as noted).

| **Scientific name** | **Common name** | **Driver** |
| --- | --- | --- |
| Sassafras albidum | Sassafras | Climate (MS, NH, OK, TX); All scenarios (FL, LA, SC) |
| Pinus banksiana | jack pine | Climate (NE, NY); All scenarios (IL, PA) |
| Quercus velutina | black oak | Climate (TX); All scenarios (LA) |
| Robinia pseudoacacia | black locust | Deposition (LA, NH, RI); All scenarios (TX) |
| Quercus muehlenbergii | chinkapin oak | All scenarios (NC) |
| Betula papyrifera | paper birch | Deposition (OH) |

**Table S3**. Millennium Ecosystem Assessment (MEA) ecosystem service categories and types (MEA, 2005). “Species-specific services” indicates the services offered (and included in the reported “counts”) by the 94 species evaluated in this study.

**Table S4.** Summary of individual species biomass responses to reductions in N+S deposition and climate change scenarios (national level) and counts of provisioning and cultural services (summed in Table 2) offered by each species as determined from USFS FEIS (<http://www.fs.fed.us/database/feis/>). The percent increases or decreases in response to reduced deposition and climate change scenarios are the percent difference in tree species biomass (national level) relative to constant deposition (D_0_) or constant climate (CC), calculated as: (deposition or climate scenario – constant)/constant, using national-level (summed) tree biomasss averaged by deposition form and climate scenario. Cells highlighted in red indicate <-10%, yellow indicate 0 to -10%, light blue indicate 0 – 10%, and dark blue indicate >10% differences.

**Table S4. Continued.**

**Table S4. Continued.**

**Table S4. Continued.**

**Table S4. Continued.**

**Table S4. Continued.**

**Table S5.** Example species with positive responses to reductions in N+S deposition and associated unique services offered by each species across climate scenarios. The percent increases or decreases in response to reduced deposition and climate change scenarios are the percent difference in tree species biomass (national level) relative to constant deposition (D_0_) or constant climate (CC), calculated as: (deposition or climate scenario – constant)/constant, using national-level (summed) tree biomass averaged by deposition form and climate scenario. Cells highlighted in red indicate <-10%, yellow indicate 0 to -10%, light blue indicate 0 – 10%, and dark blue indicate >10% differences. See USFS FEIS (USFS FEIS - <http://www.fs.fed.us/database/feis/>) for the references associated with the ecosystem services.

**Table S6.** Example species with negative responses to reductions N+S deposition and associated unique services offered by each species across climate scenarios. The percent increase or decreases in response to reduced deposition and climate change scenarios are the percent difference in tree species biomass (national level) relative to constant deposition (D_0_) or constant climate (CC), calculated as: (deposition or climate scenario – constant)/constant, using national-level (summed) tree biomass averaged by deposition form and climate scenario. Cells highlighted in red indicate <-10%, yellow indicate 0 to -10%, light blue indicate 0 – 10%, and dark blue indicate >10% differences. See USFS FEIS (USFS FEIS - <http://www.fs.fed.us/database/feis/>) for the references associated with the ecosystem services.

**Figure S1.** USFS National Forest Regions used to calculated species-specific sawtimber volumes and values (<https://www.fs.usda.gov/detailfull/r1/about-region/%3Fcid%3Dstelprdb5110505%26width%3Dfull>)
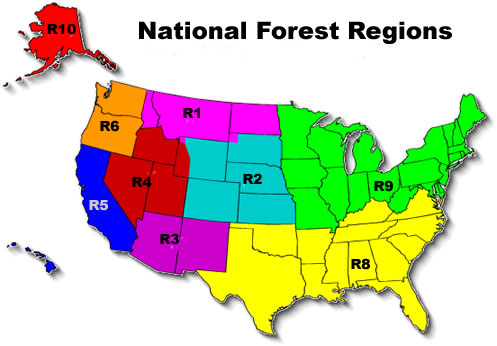
.
